# Large bilateral ovarian cysts with left ovarian torsion and right dermoid cyst

**DOI:** 10.11604/pamj.2020.37.191.26328

**Published:** 2020-10-29

**Authors:** Sara Ait Souabni, El Habib Belhaddad

**Affiliations:** 1 Faculty of Medicine and Pharmacy of Marrakech, Cadi Ayyad University, Marrakech, Morocco

**Keywords:** Torsion, dermoid cyst, case report

## Image in medicine

We report the case of a 19-year-old patient, with medical history of hypothyroidism and nulligravida, who presented with acute pelvic pain progressing for 5 days. On clinical examination, the patient was stable and had pelvic tenderness. The speculum and vaginal examination were not performed on account of the patient being a virgin. A pelvic magnetic resonance imaging (MRI) was performed showing a cystic right ovarian lesion measuring 9.7 x 7 x 6 cm of benign appearance with swollen ovary probably related to a torsion; associated with a left ovarian lesion measuring 10 x 10 x 6 cm with a double cystic and fatty component in favor of a dermoid cyst (A,B,C). Surgical exploration showed two large bilateral ovarian cysts (D). The right ovary was the site of 3-whirl torsional ischemia. The left ovary presented with a large cyst with a fatty and fluid cartilaginous component (E). The uterus was unicorn with the presence of a rudimentary straight horn. The operative procedure consisted of a bilateral cystectomy with right salpingectomy in order to avoid an ectopic pregnancy on a rudimentary horn. The right ovary revascularized after untwisting and the postoperative course was normal.

**Figure 1:**
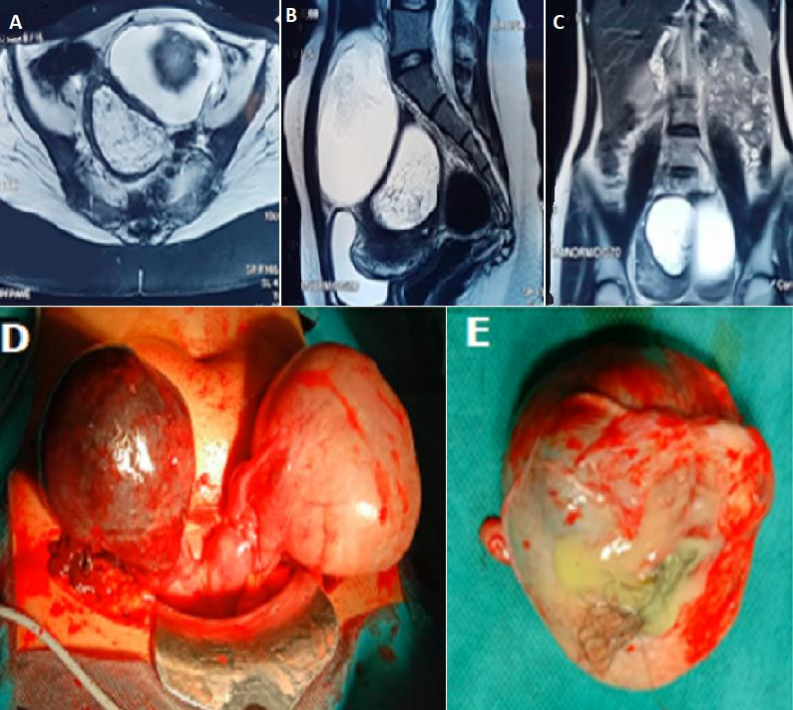
A) transverse pelvic MRI showing two bilateral ovarian cystics with torsion on the right and aspect of dermoid cyst on the left; B) sagittal pelvic MRI showing two bilateral ovarian cystics with torsion on the right and aspect of dermoid cyst on the left; C) frontal pelvic MRI showing two bilateral ovarian cystics with torsion on the right and aspect of dermoid cyst on the left; D) two large bilateral ovarian cysts with right ovarian ischemia and left dermoid cyst; E) left dermoid cyst after cystectomy

